# Emission and performance analysis of diesel engine running with CeO_2_ nanoparticle additive blended into castor oil biodiesel as a substitute fuel

**DOI:** 10.1038/s41598-024-58420-0

**Published:** 2024-04-01

**Authors:** Samuel Tamrat, Venkata Ramayya Ancha, Rajendiran Gopal, Ramesh Babu Nallamothu, Yared Seifu

**Affiliations:** 1 Department of Mechanical Engineering, Adama Science and Technology University, Adama, Ethiopia; 2 Department of Mechanical Engineering, Jimma University, Jimma, Ethiopia; 3 Department of Mechanical Engineering, Bahir Dar University, Bahir Dar, Ethiopia; 4 School Mechanical and Industrial Engineering, DDU, Dire Dawa, Ethiopia

**Keywords:** Performance, Emission, CeO_2_, Biodiesel, Mechanical engineering, Materials for energy and catalysis

## Abstract

The implications of adding cerium oxide (CeO_2_) nanoparticles as a fuel additive to a castor oil biodiesel–diesel fuel blend on engine performance and emissions in a single-cylinder four-stroke diesel engine under various speed were examined in the current study. The test fuels used were fossil diesel fuels, B5 blend biodiesel (as 5% biodiesel and 95% diesel), B10 blend biodiesel (as 10% biodiesel and 90% diesel), B15 blend biodiesel (as 15% biodiesel and 85% diesel), B20 blend biodiesel (as 20% biodiesel and 80% diesel), and B25 blend biodiesel (as 25% biodiesel and 75% diesel), with cerium oxide (CeO_2_) nanoparticle additive (75 ppm). The result of the physio-chemical properties of the oil samples was within the limit of the ASTM standard. The addition of CeO_2_ nano additive to the biodiesel–diesel blends has demonstrated a significant reduction in emission and increased in engine performance for all biodiesel–diesel blends for the engine operating speed range. From the result B25 have the maximum reduction rate in BSFC and B10 have the minimum reduction rate in BSFC. The average maximum increment of thermal efficiency was 22.2% for B10 with CeO_2_ inclusion. CO emission increased as engine speed increased. HC emission was reduced for all blend, with and without CeO_2_ nano additions as speed increased. Maximum NO_x_ emission was seen at the rated speed of 2700 rpm without nano additive and at 2900 rpm with nano additive. CeO_2_ nano additive reduced the soot opacity by 11.56% for all biodiesel–diesel blends for the engine operating speed range. As the objective of this study the results indicates CeO_2_ nano additive reduced emissions and improved the performance. So, using sustainable biodiesel–diesel blends made from castor oil with CeO_2_ nano additive advisable in ideal operating conditions for diesel engines.

## Introduction

The world's energy demand occasionally increases significantly due to population growth, the development of new infrastructure, and technological developments^1,2^. Diesel engines have higher braking thermal efficiency than gasoline engines and use less fossil fuel. Petroleum diesel is used extensively in a variety of industries. However, overall petroleum reserves are dwindling on a daily basis as a result of indiscriminate extraction, extravagant consumption and massive emissions^3–6^. Numerous academics have been compelled to look for alternate energy alternatives due to the global energy demand^7–9^. Fossil fuels like coal or petroleum are currently used to meet this increasing energy demand^10–12^. As a result, the researchers believe that biodiesels are a feasible alternative to diesel fuel in diesel engines. Biodiesel can be made from a variety of renewable feedstocks, including edible oils such as palm oil, sunflower oil, and peanut oil, as well as non-edible oils such as castor, jatropha, cotton seed, and waste plastic oil^3,13–15^.

Castor (*Ricinus communis* L.) is non edible oilseed crop adapted to dry lands of tropics and semi-arid tropics. In Ethiopia, Castor grows as annual in the low lands to small tree perennial in the high lands^16^. Vegetable oils are the most commonly used feedstocks in the extraction and production of biodiesel. Among these, castor oil offers two intriguing properties as a raw material for biodiesel: it doesn't compete with cooking oils and its growth doesn't require a lot of inputs^17^.

Among the different strategies available to reduce exhaust emissions, the use of fuel-borne catalysts is now being prioritized due to the benefits of increased fuel efficiency while lowering hazardous greenhouse gas emissions and health-threatening compounds^18–21^. Recent research focused on alternative fuels and additives to improve engine performance and reduce emissions^22,23^. CeO_2_ nanoparticles promote combustion temperature at the time of reactions with oxygen and the engine power output is improved^24,25^. Due to their similar characteristics to those of diesel, alternative fuels, in particular biodiesel and different additives, could eventually replace them^26,27^.

Based on recent literature, the most common performance property was investigated by varying percentages of biodiesel and by adding different percentages of nanoparticle additives. Because biodiesel has a lower volatility and a higher viscosity, its highest cylinder pressure decreases as the amount of biodiesel in the blend increases^28,29^. The use of Al_2_O_3_, CeO_2_ and copper oxide as an additive increased cylinder pressure because those additives have oxygen in its molecule in addition to the oxygenated biodiesel, which accelerates the combustion process that leads to increased power and torque output^30,31^. Because the metal-based nano additions are oxygenated, they raise the cetane number. Due to its oxygen content, one of these, the well-known metal oxide nano-additive cerium oxide, displays a notable redox response. Its reaction with carbon atoms, HC molecules, and CO molecules in soot produces large amounts of oxygen, allowing the fuel to burn entirely^32^.

Vegetable oils have less sulfur content, high cetane numbers, are highly oxygenated, have greater combustion efficiency, and a less amount of emission when they mix with the nano particles^33^. According to recent research findings, nanoparticles can be used as an inventive diesel addition to enhance engine efficiency. Studies using a single diesel engine and a diesel–biodiesel mixture made from castor that has high CO and HC emissions are what led to the research gap in this area. As a result, it is critical to evaluate castor diesel–biodiesel blends with 75 ppm CeO_2_ nanoparticles to minimize emissions and improve engine performance. This study's objectives include reducing emissions, using sustainable biodiesel–diesel blends made from castor oil, and determining the ideal operating conditions for diesel engines.

The novel idea of the present work lies in producing castor biodiesel by the optimization process and mixing with CeO_2_ nanoparticles and formulating the nano-based bi-compound fuel additive to improve the fuel quality, engine functionality, and reduction of harmful exhaust emissions from diesel engine. The present investigation witnessed the ability of nano particles in boosting up the engine combustion performance and reduction of harmful emissions. The other novel finding of this research work was, the engine rated speed where the maximum torque achieved were at 2700 rpm. Hence, the rated engine speed is determined by the type of fuel blends and additives.

## Materials and methods

### Extraction of oil and preparation of biodiesel

**Figure 1:**
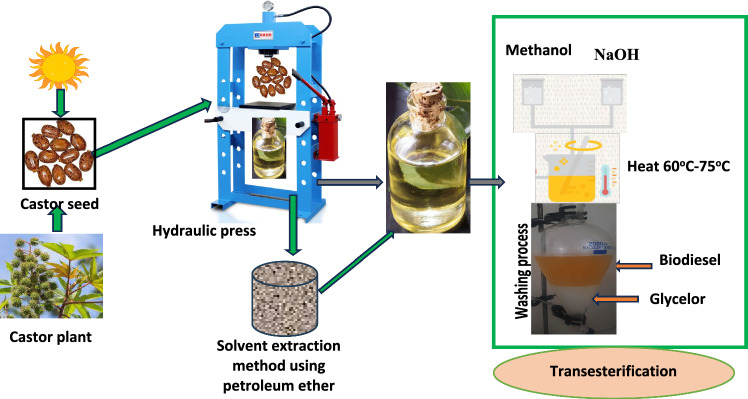
Procedures for Extraction of oil and Preparation of biodiesel.

In this research work, Castor seeds were used, as a feedstock for the synthesis of biodiesel, which were bought from farmers of the local market. The extraction process employed were solvent method and hydraulic press as shown in Fig. 1. During solvent method of extraction seeds were crushed, and soaked for forty-eight hours after stirred frequently and wait for some time to settle then filter paper was used for filtering to separate from the residue. For the bulk production of oil, a hydraulic press was used. During the extraction oil from 34 kg of castor seed the yield was 12.5 L. In this study castor seed have yield of 367.6 ml/kg. During the extraction process of the oil, the yield was 28% (v/w) and 51% (v/w) for the solvent method and hydraulic press method respectively.

Before the synthesis of the biodiesel from the parent oil of Castor seeds, it was mandatory to determine the free fatty acid (FFA) whether the process has to undergo through esterification or transesterification reaction. The parent oil’s acid value was 4.6% that could lead to saponification. During the transesterification reaction, NaOH and analytical-grade methanol were used as seen in Fig. 1.

The molar ratio of the alcohol to the oil was 1:5 and 0.8% volume of sulfur acid was added while the hotplate’s speed and temperature were set to 800 rpm and 65 °C respectively for 70 min. At this stage, the acid value was lowered from 4.6 to 0.8 mg-KOH/g-oil. The second stage was the transesterification process by using base catalyst sodium hydroxide and methanol that dissolved to form metha-oxilate at which the ratio was 25-g NaOH to one litter methanol. The methyl ester was washed three times to remove the soap. The removal of the moisture content of the biodiesel was done using a furnace at a temperature of 130 °C. The properties of the oil of castor after esterification were characterized as per the ASTM 6571 standard to qualified the oil samples as biodiesel.

### Experimental procedure and setup

After oil samples are qualified as biodiesel based on the ASTM 6571 standard the inclusion of CeO_2_ in biodiesel–diesel blend were done. The size of the nano additive CeO_2_ for the biodiesel–diesel mix was 25 nm, which was purchased from commercially available in Sigma Aldrich level with the density of 7.13 gm/ml with 100% purity level. During the inclusion of the nanoparticles, ultra-sonication was executed for 1 h at a frequency of 90 kHz for the proper dispersion of the nanoparticles. The prepared fuel samples were B0, B5, B10, B15_,_ and B25 with 75 ppm CeO_2_ nano additive. The reason why 75 ppm nano additive CeO_2_ was used is because, a pilot test was executed by using doses of 55, 65, 75, 90 and 100 ppm. However, a slight reduction of the UHC and NO_X_ was seen as the dose increases and it was stable at 75 ppm. Therefore, 75 ppm dose was selected for this experimental research work.

**Figure 2:**
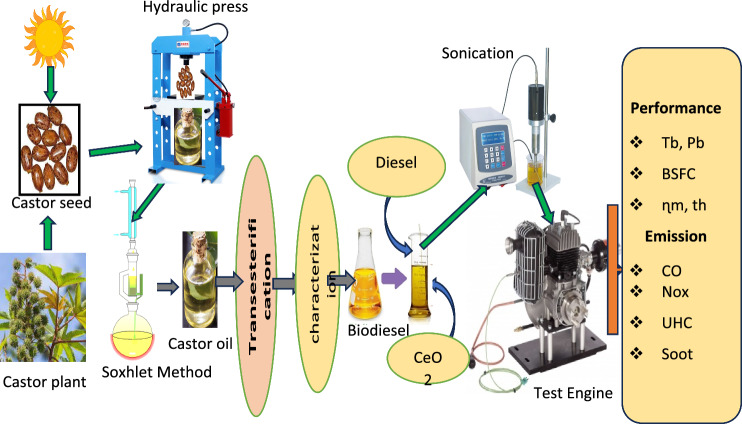
Over all experimental Procedures.

The overall procedure done during this research work starting from castor seed collection to experimental test on engine test rig is indicated in Fig. 2.

**Figure 3:**
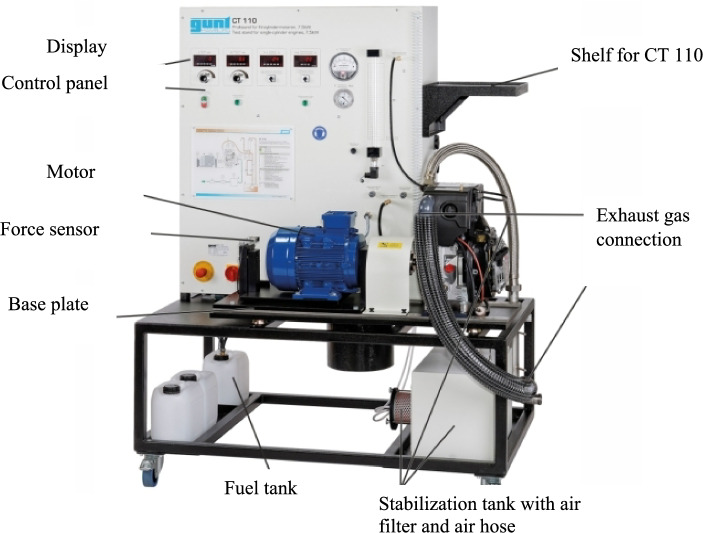
Experimental setup.

In this study, the fuel samples were investigated using the test setup unit, Gunt model CT110, as shown in the experimental setup in Fig. 3. The test stand consists of a CT110 engine mount, dynamometer, and CT110.22 diesel engine. The main function of the engine mount is to record and display data of experiments on the dashboard. The engine used for the performance test is a naturally aspirated mono-cylinder diesel engine. The test engine is started by, the asynchronous motor equipped with the exhaust gas temperature sensor. The fuel tank, the vessel for the intake of air, and the fuel flow meter are fixed at the mobile frame. The fuel samples were tested at 80% load ranging from 1600 to 3000 rpm. The results of the experiment were displayed by the data acquisition system on a personal computer. Additionally, in each case, the exhaust gas analyzer shows the emission results like the HC, CO, and NO_x_ at their respective speed of measurement.

For emissions measurement, exhaust gas analyser placed on the line of the engine exhaust gas was used to analyse the main pollutants (HC, CO_2_, CO, O_2_, NOx) measured by Gunt, CT159.02 digital analyser. The detail specification of the exhaust gas analyser indicated in Table 1.

**Table 1:** Exhaust gas analyzer specification.

Parameters	Specification
Nominal/Power	0.2 KW
Type	Gunt, CT159.02
Volt	230 V
Frequency	50 Hz
Fabrication number	237,653

The combustion and performance characteristics have been performed on CT 110 single cylinder engine. The detail specification of the test engine explored in Table 2.

**Table 2:** Specification of the test engine.

Engine parameters	Specification
Engine Model	EA300-E2-NB1
Type of stoke	Four-stroke diesel engine
Cooling system	Water-cooled
Cylinder arrangement	Single cylinder
Maximum power	7.5 kW@3000 min-1
Engine displacement	309 cm^3^
Bore x stoke	75 mm × 70 mm
Compression ratio	23:1
Oil capacity	1,3 L
Noise level	95 dB(A)
Rotameter	30…300 L/h
Temperature sensor, exhaust gas temperature	0…1000 °C
Type of ignition	Compression ignition
Rod length	114.5 mm
Crank length	34.5 mm

### Uncertainty analysis

The highest mean percentage uncertainty (Ūmax) predicted for engine exhaust emissions and engine combustion performance is shown by the accuracy connected with the measuring instruments, as shown in Table 3.

**Table 3:** Accuracy and uncertainties.

Measured parameters	Instrument	Measuring range	Accuracy%	Ū_max_ (%)
NO_x_	Kane AUTO plus gas analyzer	0–5000 ppm	± 12 PPM	17.5
CO	CT159.02 Exhaust gas analyzer	0–10% vol	± 0.06% vol	8.7
HC		0–2500 ppm	± 3 ppm	6.2
Speed	CT 110.20	0–5000 rpm	± 12 rpm	0.51
Brake Power	CT 110.20	0–7.5 kW	± 0.1	2.72
Brake Torque	CT 110.20	− 50–50 Nm	± 0.2	1.96
TFC	CT 110.20	50 cm^3^/min	± 0.05	3.04

## Result and discussion

The baseline diesel fuel and the biodiesel–diesel blended fuel properties were measured, and analyzed in this study. It revealed that all the blended castor oil biodiesel–diesel fuel satisfied the standard requirement of ASTM D6571 specifications. The result of the Physio-chemical properties of biodiesel–diesel blends are summarized in Table 4. The result of the physio-chemical properties of the oil samples was within the limit of the ASTM standard requirement of fuel for diesel engine propulsion. The flash point of biodiesels was 79.1 °C, 76.7 °C, 75.5 °C and 73.4 °C respectively for B25, B15, B10_,_ and B5, which is higher than that of pure petro diesel of 72.3 °C. Therefore, biodiesel is safer to fire hazard during storage and custody transfer of fuel. The cloud point, of biodiesel was less than 0 °C, which is higher than pure diesel fuel with a cloud point of 0 °C. Hence, the biodiesel produced from castor has to be used in a hot climate so additives should be added to lowers the freezing point of the fuel. The biodiesel produced from castor was safer for storage and handling because biodiesel is denser than petro diesel, as indicated on Table 4. The boiling point of the biodiesel has a higher initial boiling point that affects starting of engines at lower temperature and which causes higher fuel consumption during starting. Moreover, at lower engine speeds the unburned hydrocarbon emission due to this property.

**Table 4:** Physio-chemical properties of diesel and biodiesel blends.

NO	Physio-chemical properties	ASTM	Limit	Test result of blends				
			6751-07b	B25	B15	B10	B5	B0
1	Density@15 °C , g/ml	D1298	Report	0.875	0.862	0.857	0.8525	0.85
2	Density@20 °C , g/l	D1298	Report	0.861	0.856	0.852	0.85	0.84
3	Flashpoint (°C)	D93	Max. 100	79.1	76.7	75.5	73.4	72.3
5	Cloud point, °C	D2500	Report	0	− 1	− 1	− 1	0
6	Pour point, °C	D97	Report	< − 8	< − 8	< − 8	< − 8	< − 8
7	Kinematic viscosity	D445	1.9—6	3.828	3.5116	3.2865	3.2013	3.1637
8	Cetain Index	D976	Min. 47	51.34	51.127	47.56	50.46	52.905
9	ASTM color	D1500	Max. 3	1 < x < 1.6	1 < x < 1.6	1 < x < 1.6	1 < x < 1.6	1
10	Water & segment, %V	D2709	Max. 0.03	< 0.03	< 0.03	< 0.03	< 0.03	< 0.03
11	Acidity, mg KOH/g	D974	0.5	0.105	0.048	0.0308	0.02421	0.0112
12	Ash content, mass%	D482	Max. 0.01	0.0009	0.0005	0.0004	0.0003	0.0002
13	Calorific value, Cal/g	–	Report	10,510	10,599	10,678	10,790	11,200

### Engine performance

Engine performance tests was conducted using the blended fuels B0, B5, B10, B15_,_ and B25 with 75 ppm CeO_2_ nano additive and without nano additive for comparison. All the engine tests were conducted from the speed range of 1600 rpm up to 3000 rpm at 80% load.

**Figure 4:**
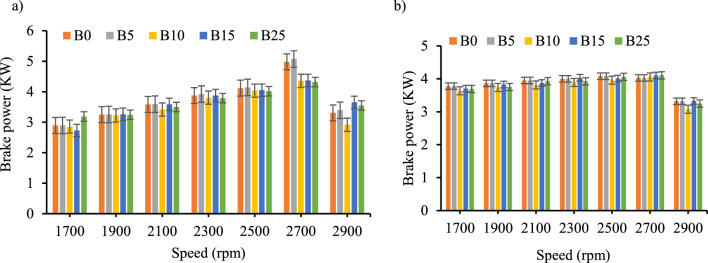
Brake power versus engine speed for all blend ratios. (**a**) Brake power with out CeO_2_ Nano particle, (**b**) Brake power with CeO_2_ Nano particle.

*Brake power *Engine's brake power was evaluated with diesel and biodiesel–diesel blends with and without the inclusion of CeO_2_. The brake power showed a linear and insignificant increment as the ratio of the biodiesel in the blends increased. From the Fig. 4a and b, the break power reduced slightly from the baseline fuel performance except for B25 because of the biodiesel blends have lower calorific value and higher viscosity as seen on Table 4. Similarly, other researchers showed the same outcome^34–36^.

The result of the brake power output of the test engine with the inclusion of CeO_2_ showed, an average increment of 0.15 kW, 0.12 kW, 0.18 kW, 0.19 kW, and, 0.09 kW for B0, B5, B10, B15_,_ and B25 respectively. Moreover, the maximum power output was at rated speed of 2700 rpm for both cases. As shown on the plot in Fig. 4a and b, the inclusion of CeO_2_ nanoparticles have shown a smooth power output in a wide speed operating range decreased at maximum speed.

*Brake torque* The torque output of this study for the blends with and without CeO_2_ nanoparticles has shown similar patterns. However, the inclusion of CeO_2_ additives on the diesel–biodiesel blends indicated a significant increment of engine torque for the blend B25 especially at the engine speed of 1700-rpm.

**Figure 5:**
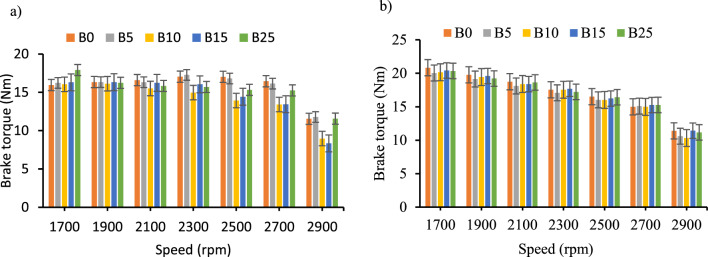
Brake torque versus engine speed for all blend ratios. (**a**) Brake torque with out CeO_2_ Nano particle, (**b**) Brake torque with CeO_2_ Nano particle.

The brake torque output of the test engine with the inclusion of CeO_2_ showed, average increment of 1Nm, 0.6Nm, 2.4Nm, 2.5 Nm, and 1.3Nm for B0, B5, B10, B15_,_ and B25 respectively. Moreover, the maximum torque output was recorded at lower engine speed. As shown on the Fig. 5a and b, the inclusion of CeO_2_ nanoparticles has shown a smooth torque output in a wide speed decreased as engine speed increased. Recent studies also reported similar results^23,30,31,37^.

**Figure 6:**
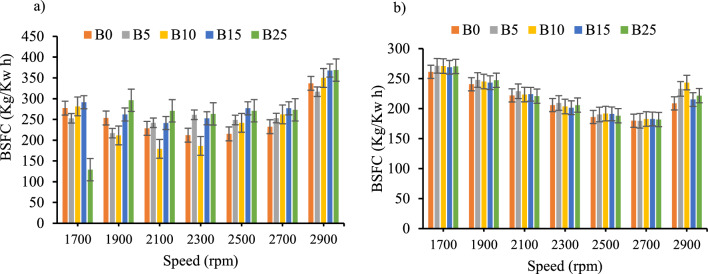
BSFC versus engine speed for all blend ratios. (**a**) BSFC with out CeO_2_ Nano particle, (**b**) BSFC with CeO_2_ Nano particle.

*Brake-specific fuel consumption *Fig. 6a and b shows, the effect of BSFC as a function of engine speed and biodiesel–diesel blends with and without CeO_2_ nano-additives respectively. The result of the study showed that the BSFC for all the blends with and without CeO_2_ were minimum at the intermediate engine speed which is the economic speed range of the test engine. However, in this speed range, the BSFC of the biodiesel–diesel blends with CeO_2_ inclusion have shown a reduction of 16.71%, 16.32%, 12.01%, 23.14%, and 26.02%, for B0, B5, B10, B15_,_ and B25 respectively. From the result B25 have the maximum reduction rate in BSFC and B10 have the minimum reduction rate in BSFC. Previous study has shown the higher percentage of biodiesel have higher reduction rate of BSFC when CeO_2_ nano additives are mixed with biodiesel^38,39^. Moreover, the fuel consumption reduced as the biodiesel ratio increased in the blends as shown in Fig. 6a and this is similar to a previous study conducted by^40,41^.

**Figure 7:**
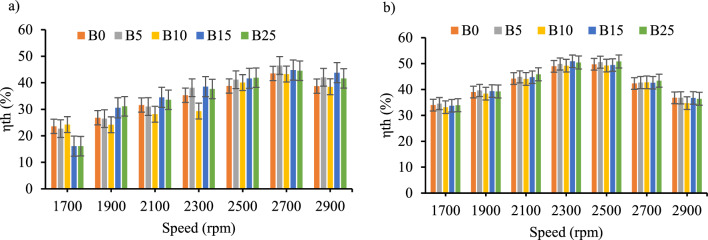
Thermal efficiency versus engine speed for all blend ratios. (**a**) ɳ_th_ with out CeO_2_ Nano particle, (**b**) ɳ_th_ with CeO_2_ Nano particle.

*Brake thermal efficiency* Across the engine operating speed range, B15 and B25 had higher engine thermal efficiency as shown in Fig. 7a except at engine speed of 2700 rpm. According to a prior study, using castor biodiesel boosts brake thermal efficiency when compared to diesel fuel^40^. As seen in prior research, the thermal efficiency increased as the fraction of biodiesel in the blends increased^41–44^. The brake thermal efficiency with the inclusion of CeO_2_ nanoparticles showed the maximum increment specifically at lower engine speed as shown in Fig. 7a and b. The average maximum increment of thermal efficiency was 22.2% for B10 with CeO_2_ inclusion. The performance of internal combustion engines has been demonstrated to be significantly impacted by cerium oxide^32^. These additives work as a catalyst, bringing oxygen to the fuel mixture to speed up combustion and increase engine efficiency overall.

**Figure 8:**
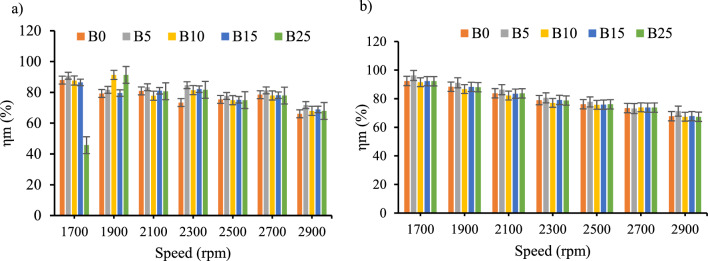
Mechanical efficiency versus engine speed for all blend ratios. (**a**) ɳ_m_ with out CeO_2_ Nano particle, (**b**) ɳ_m_ with CeO_2_ Nano particle.

*Mechanical efficiency* As indicated on the Fig. 8a and b the mechanical efficiency decreased as the engine speed increased for both with and without CeO_2_ nanoparticles. However, the maximum increment of mechanical efficiency of the biodiesel–diesel blends with CeO_2_ inclusion was 3.2% and 3.6%, for B15 and B25 respectively. The decrement of mechanical efficiency with the engine speed were in line with earlier investigations^23,41,45^.

### Emission analysis

**Figure 9:**
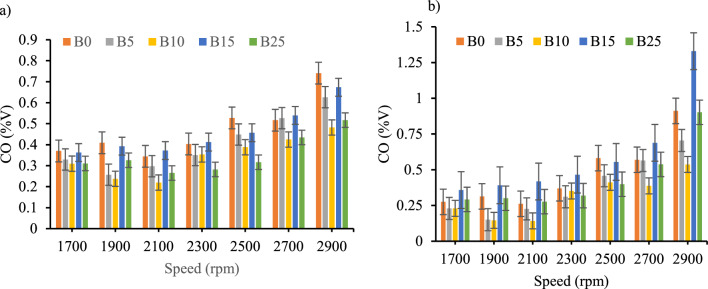
Carbon monoxide emission versus engine speed for all blend ratios. (**a**) CO Emission with out CeO_2_ Nano particle, (**b**) CO Emission with CeO_2_ Nano particle.

*Carbon monoxide emission* Carbon monoxide **(**CO) emission was generally lower than diesel fuel as shown in Fig. 9a in case where nano additive is not used from the rated idle speed to the rated maximum speed. Addition of CeO_2_ nano additive to biodiesel–diesel blends increase the CO emission as engine speed increased as shown on Fig. 9b. When CeO_2_ nanoparticles were added, the maximum average percentage reduction of CO emission was 12% and 10.2% for B5 and B10 respectively. Whereas for the blends of B0, B15_,_ and B25, CO emission was increased by 1.5%, 30% and 27% respectively. The increment of the CO emission with engine speed were in line with earlier investigations^41,46–48^.

**Figure 10:**
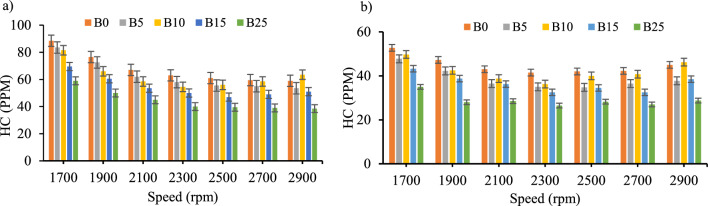
Unburned hydrocarbons emission versus engine speed for all blend ratios. (**a**) HC Emission with out CeO_2_ Nano particle, (**b**) HC Emission with CeO_2_ Nano particle.

*Unburned hydrocarbon emission* The relationship between engine speed and hydrocarbon (HC) emissions is shown in Fig. 10a and b for blends with and without CeO_2_ nano-additives. Except for B10 at the highest engine speed, the emission of HC reduced as the proportion of biodiesel increased shown in Fig. 10a. Additionally, HC emission was reduced for all blend, with and without CeO_2_ nano additions as speed increased. Due to the higher cetane number, higher oxygen content biodiesel by itself, and inclusion of CeO_2_ in the biodiesel–diesel blends, the formation of HC emission in the engine cylinder is minimized^9,12,49,50^. The incorporation of CeO_2_ nanoparticles showed an average percentage reduction in HC emission was 32.3, 37.7, 32.6, 33 and 32.8% for B0, B5, B10, B15_,_ and B25, respectively. The percentage of reduction of HC emission when nano-additives used were also the same with the earlier research works^40,41,51^.

**Figure 11:**
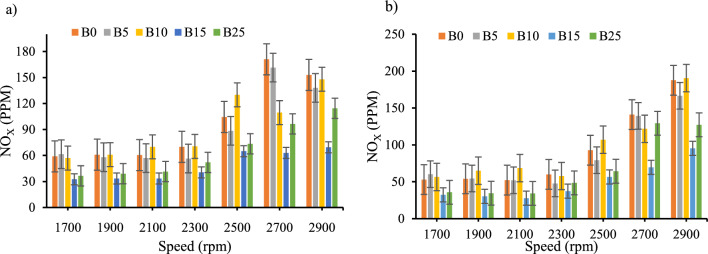
Nitrogen oxide emission versus engine speed for all blend ratios. (**a**) NOx Emission with out CeO_2_ Nano particle, (**b**) NOx Emission with CeO_2_ Nano particle.

*Nitrogen oxide emission *Fig. 11a and b depict the emissions of nitrogen oxide emission (NOx) to speed for biodiesel–diesel blends without and with the inclusion of CeO_2_ nano additions, respectively. Maximum NO_x_ emission was seen at the rated speed of 2700 rpm without nano additive and at 2900 rpm with the CeO_2_, where maximum power and torque are obtained. The trend of NO_x_ emissions increased as engine speed increase. In addition, the inclusion of nano additive CeO_2_ reduced NO_x_ emissions by 5.5% and 2.4% for B0 and B5 respectively. Whereas for the blends of B10, B15_,_ and B25, NO_x_ emission was increased by 4.3%, 3.3% and 8.5% respectively. This percentage reduction of NO_x_ emission when nano-additives incorporated were also the reported by earlier research works (Agarwal et al., 2015)^23,51,52^.

**Figure 12:**
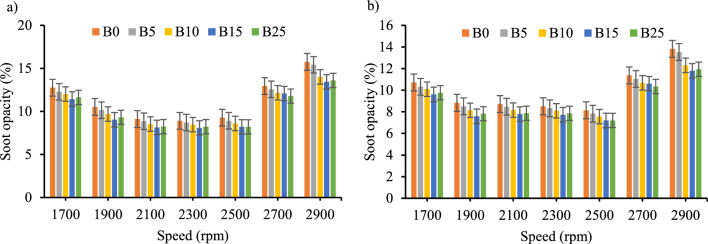
Soot opacity (%) versus engine speed for all blend ratios. (**a**) Soot opacity (%) without CeO_2_ Nano particle, (**b**) Soot opacity (%) with CeO_2_ Nano particle.

*Soot opacity* The experiments result of smoke opacity are shown in Fig. 12a and b for both biodiesel–diesel blends with and without CeO_2_ respectively. Averagely the use of CeO_2_ nano-additives showed less than 12% smoke opacity. Biodiesel–diesel blends have demonstrated soot opacity of less than 10% between 1900 and 2600 rpm.

The fuel sample that had the greatest and the lowest amount of smoke opacity was B0 and B25 respectively through engine speeds. However, the addition of CeO_2_ nano additive to the biodiesel–diesel blends has demonstrated a significant average reduction in soot opacity of 11.56% for all biodiesel–diesel blends for the engine operating speed range. This conclusion was consistent with the study's findings^41,45,52–54^.

## Conclusion

The current study investigates the effects of CeO_2_ nanoparticles on four fuel mixes running in a single-cylinder DI diesel engine at dose levels of 75 ppm for investigation of performance and emission. This study has shown a rise in brake thermal efficiency with the inclusion of CeO_2_ nanoparticles. Using CeO_2_ nano-additives decreased the output of CO emissions for all biodiesel–diesel blends at all engine speeds. Due to the higher Cetane number and oxygen content of the biodiesel and the nano additions, the emission of HC in the engine cylinder was decreased as CeO_2_ nanoparticles were added to the diesel blends. The addition of CeO_2_ demonstrated a considerable reduction of NO_x_ emission and soot opacity for all biodiesel–diesel blends for the engine operating speed range. By adjusting the blend ratio and engine speed with and without CeO_2_, at a fixed compression ratio and 80% load, this study investigated the performance and emission characteristics of castor biodiesel–diesel blends. According to the results, B25 has the highest BSFC reduction rate and B10 has the lowest. For B10 with CeO_2_ addition, the average highest improvement in thermal efficiency was 22.2%. As engine speed increased, so did CO emissions. As speed increased HC emission decreased for all blends, both with and without CeO_2_ nano additions. Maximum NOx emission was observed at 2900 rpm with nano additive and at the rated speed of 2700 rpm without it. Throughout the engine operating speed range, the CeO_2_ nano addition decreased the soot opacity by 11.56% for all biodiesel–diesel mixes. In general, CeO2 improves the properties of mixes of biodiesel. Additionally, it enhances the diesel engine's biodiesel blend's combustion efficiency and reduces pollutants. It is necessary to design new, modified injection strategies with several injection events to mitigate the increase in NOx emissions. Consequently, we suggest that further research be done to investigate the performance, combustion, and emission characteristics of diesel engines by varying the injection timing, compression ratio, and injection rate.

## Data Availability

Upon reasonable request, the corresponding author will provide the data supporting the study conclusions.

## List of symbols

ASTM: American society for testing and materials
B10: 10% Biodiesel and 90% diesel
B15: 15% Biodiesel and 85% diesel
B20: 20% Biodiesel and 80% diesel
B25: 25% Biodiesel and 75% diesel
B5: 5% Biodiesel and 95% diesel
BSFC: Brake specific fuel consumption
BTE: Brake thermal efficiency
CeO_2_: Cerium oxide
CI: Compression ignition
CO: Carbon monoxide
CO_2_: Carbon dioxide
DI: Direct injection
FFA: Free fatty acid
HC: Hydrocarbons
NaOH: Sodium hydroxide
NO_X_: Nitrogen oxides
UHC: Unburned hydrocarbons
